# Dimethylarginine dimethylaminohydrolase 2 promotes tumor angiogenesis in lung adenocarcinoma

**DOI:** 10.1007/s00428-015-1863-z

**Published:** 2015-10-29

**Authors:** Toshihiro Shiozawa, Shinji Iyama, Shotaro Toshima, Akiko Sakata, Shingo Usui, Yuko Minami, Yukio Sato, Nobuyuki Hizawa, Masayuki Noguchi

**Affiliations:** Department of Pathology, Faculty of Medicine, University of Tsukuba, 1-1-1 Tennodai, Tsukuba, Ibaraki 305-8575 Japan; Department of Pulmonary Medicine, Faculty of Medicine, University of Tsukuba, Tsukuba, Ibaraki Japan; Department of Thoracic Surgery, NHO Ibarakihigashi National Hospital, Tokai, Ibaraki Japan; Department of Thoracic Surgery, Faculty of Medicine, University of Tsukuba, Tsukuba, Ibaraki Japan

**Keywords:** DDAH2, Angiogenesis, Adenocarcinoma, Malignant stroma, Prognosis

## Abstract

Although embryonal proteins have been used as tumor marker, most are not useful for detection of early malignancy. In the present study, we developed mouse monoclonal antibodies against fetal lung of miniature swine, and screened them to find an embryonal protein that is produced at the early stage of malignancy, focusing on lung adenocarcinoma. We found an antibody clone that specifically stained stroma of lung adenocarcinoma. LC-MS/MS identified the protein recognized by this clone as dimethylarginine dimethylaminohydrolase 2 (DDAH2), an enzyme known for antiatherosclerotic activity. DDAH2 was found to be expressed in fibroblasts of stroma of malignancies, with higher expression in minimally invasive adenocarcinoma (MIA) and invasive adenocarcinoma than in adenocarcinoma in situ (AIS). Moreover, tumors with high stromal expression of DDAH2 had a poorer prognosis than those without. In vitro analysis showed that DDAH2 increases expression of endothelial nitric oxide synthase (eNOS), inducing proliferation and capillary-like tube formation of vascular endothelial cells. In resected human tissues, eNOS also showed higher expression in invasive adenocarcinoma than in AIS and normal lung, similarly to DDAH2. Our data indicate that expression of DDAH2 is associated with invasiveness of lung adenocarcinoma via tumor angiogenesis. DDAH2 expression might be a prognostic factor in lung adenocarcinoma.

**Electronic supplementary material:**

The online version of this article (doi:10.1007/s00428-015-1863-z) contains supplementary material, which is available to authorized users.

## Introduction

Lung cancer is the leading cause of cancer-related death worldwide. Because most patients are diagnosed at an advanced stage, the prognosis of lung cancer remains poor. Lung adenocarcinoma is the most frequent histological type of lung cancer in Japan and is increasing in the USA and most European countries [1, 2].

Recently, a multidisciplinary classification of lung adenocarcinoma has been published, in which several new concepts, such as adenocarcinoma in situ (AIS) and minimally invasive adenocarcinoma (MIA), have been newly defined [3, 4]. AIS shows replacement growth of tumor cells along alveolar structures (lepidic growth) and contains no invasive component. Although MIA has been added to the invasive adenocarcinoma category, its area of invasion is very limited and it is considered as very early stage adenocarcinoma. Therefore, it has been suggested that MIA has a prognosis as favorable as that of AIS. AIS and MIA are usually detected accidentally or by CT screening, and it is assumed that their biological characteristics are different from those of overt invasive adenocarcinoma. The current classification has focused interest on the concept of stepwise progression of peripheral type adenocarcinoma [5]. It is very important to compare the biological features of AIS and MIA with those of overt invasive adenocarcinoma, in order to clarify molecular mechanisms involved in malignant progression of lung adenocarcinoma.

With regard to the detection and diagnosis of lung adenocarcinoma by immunohistochemistry, various tumor biomarker proteins have been reported. These biomarker proteins are classified into several types according to their characteristics and include oncogenic, tissue- and/or cell-specific, and embryonic proteins. Epidermal growth factor receptor (EGFR), Her2, B-raf, Ret, Ros and anaplastic lymphoma kinase (ALK) are examples of oncogenic biomarkers associated with prognosis [6–8]. The expression status of these oncogenes also has an important bearing on the selection of targeted therapeutic drugs, since various types of tyrosine kinase inhibitors are available. On the other hand, tissue- and/or cell-specific biomarkers include cyto-keratins (AE1/AE3, CK7, CK20, etc.), membrane markers of lymphocytes (CD20, CD79, CD3, CD4, CD8, CD9, etc.) and differentiation-specific markers such as TTF-1 for alveolar epithelial differentiation. Although tissue- and/or cell-specific biomarkers are suitable for differential diagnosis between cancer and lymphoma, or between lung adenocarcinoma and metastatic adenocarcinoma, they are not useful as indicators of malignancy.

Although it is noteworthy that these tumor biomarkers include embryonic and fetal proteins such as carcinoembryonic antigen (CEA), OCT3/4, and SOX2 [9–11], few attempts have been made to search for fetal biomarkers systematically. Experimental use of human fetal tissue has various associated ethical issues. Fortunately, most antibodies against swine tissue cross-react with human tissue, since swine messenger RNA (mRNA) has more than 80 % homology with its human counterpart. In view of this homology, fetal swine tissue constitutes an ideal antigenic material for producing antibodies reactive with human fetal tissue and human cancer tissue as embryonic biomarker.

In the present study, we immunized mice against fetal swine tissue and produced monoclonal antibodies (mAbs) reactive with human lung adenocarcinoma. One reacted characteristically with the stromal region of human lung adenocarcinoma, and the antigen was identified as dimethylarginine dimethylaminohydrolase 2 (DDAH2) by LC/MS-MS analysis. We demonstrate that DDAH2 is a marker of tumor angiogenesis and is expressed in lung adenocarcinoma at an early stage.

## Materials and methods

### Patient selection

We selected 133 cases of lung adenocarcinoma that had been resected at Tsukuba University Hospital (Ibaraki, Japan) between 2002 and 2013. All patients had given informed consent for study of their materials before collection. The adenocarcinomas were classified according to the World Health Organization (WHO) classification (fourth edition) and the UICC TNM classification of malignant tumors (seventh edition) [12]. The patients’ clinicopathological characteristics are summarized in Supplementary Table 1.

### Production of monoclonal antibodies

The normal length of gestation period in swine is approximately 16 weeks. So, we selected normal fetuses of CLAWN strain miniature swine (Japan Farm CLAWN Institute, Kagoshima, Japan) at weeks 7 and 13 of gestation, and they were harvested surgically from maternal swine. Fetal lungs were resected and embedded in Tissue-Tek OCT Compound (Sakura Finetek Japan, Tokyo, Japan), quickly frozen, and stored at −80 °C. Protein extraction was performed using T-PER^™^ Tissue Protein Extraction Reagent (Thermo Scientific, Rockford, IL) with protease inhibitor cocktail (Sigma-Aldrich, St. Louis, MO). Protein concentration was determined using a bicinchoninic acid protein assay (Thermo Scientific) with bovine serum albumin as the standard. Antigen was prepared as a mixture of extracted protein and an equivalent amount of complete Freund’s adjuvant (CFA) (Sigma-Aldrich). CFA/antigen emulsion was injected subcutaneously into the bilateral footpad of a 5-week-old BALB/c mouse on day 1, and again on day 10. On day 14, bilateral popliteal lymph node cells were harvested and fused with the murine myeloma cell line Sp2/0 using polyethylene glycol. Hybridomas were selectively cultured and cloned using the methylcellulose method in a 10 % incubator at 37 °C. Fusion, selection, and cloning were performed with a ClonaCell^®^-HY Hybridoma Cloning Kit (Stemcell Technologies, Vancouver, British Columbia, Canada) in accordance with the manufacturer’s protocol. The isolated hybridoma colonies were each suspended in individual wells of a 96-well plate.

### Antigen characterization

The immunoglobulin isotype and subtype of the selected hybridoma clone was determined using a Mouse Monoclonal Antibody Isotyping Test Kit (AbD Serotec, Kidlington, UK).

A lung adenocarcinoma cell line (Calu-3) was used for Western blotting and immunoprecipitation (IP). Calu-3 was purchased from the American Type Culture Collection (Manassas, VA). Total protein was extracted from Calu-3 using M-PER mammalian protein extraction reagent (Thermo Scientific) and from frozen tissue of surgically resected lung adenocarcinoma with T-PER. Extracted proteins were denatured at 95 °C for 5 min, separated by SDS polyacrylamide gel electrophoresis (SDS-PAGE) under reducing conditions, and transferred to PVDF membranes using an iBlot Gel Transfer System (Life Technologies Japan, Tokyo, Japan). After blocking, the membranes were incubated with purified monoclonal antibody (2 μl/ml) as the primary antibody for 1 h at room temperature. Antigen protein was detected using HRP-conjugated anti-mouse Igs (Thermo Scientific), and the protein bands were visualized with SuperSignal West Dura extended duration substrate (Thermo Scientific) and X-ray film (Kodak, Rochester, NY).

Antigen protein that had reacted with the selected monoclonal antibody was immunoprecipitated from extracted total protein of frozen lung adenocarcinoma tissue with a Co-Immunoprecipitation Kit (Thermo Scientific), in accordance with manufacturer’s protocol. Briefly, the purified monoclonal antibody was immobilized on beads in a column, and then, the extracted total protein was incubated with the beads at 4 °C for 2 h. After washing, the immunoprecipitate was eluted with acidic buffer (pH 2.8). The eluted fraction containing the antigen was denatured, separated by SDS-PAGE under reducing conditions, and stained with silver. The protein band indicating the antigen identified by matching with the Western blot was excised and cut into pieces. The excised gel piece was digested with modified porcine trypsin (Thermo Scientific), and the processed peptides were subjected to LC/MS/MS analysis with a Zaprous LC/MS system (AMR) at the National Institute for Materials Science (Tsukuba, Japan). The obtained data file was searched against the SWISS-Prot database using MASCOT MS/MS Ion Search (Matrix Science, London, UK).

### Immunohistochemistry for DDAH2 using hybridoma supernatant

All samples were fixed with 10 % formalin and embedded in paraffin. After deparaffinization, the samples were placed in blocking solution to suppress nonspecific staining for 30 min. Then, antigen retrieval was performed using citrate buffer (pH 6.0) in an autoclave at 121 °C for 15 min. The culture supernatant of the hybridoma that detected DDAH2 was used as the primary antibody, and incubation was for 60 min at room temperature, followed by incubation with the secondary antibody (EnVision kit; Dako, Tokyo, Japan) for 30 min at room temperature. Diaminobenzidine chromogen solution (Dako) was applied to the slides, followed by counterstaining with hematoxylin. Immunohistochemistry for endothelial nitric oxide synthase (eNOS) and CD31 were performed in the same way, using anti-eNOS antibody at dilution of 1:75 (ab5589, Abcam, Cambridge, UK), and anti-CD31 antibody at dilution of 1:125 (ab28364, Abcam), respectively.

The staining was judged by focusing on the tumor stroma, and normal vascular endothelium was used as a control. If an area, showing staining similar to or stronger than that in the control, exceeded 5 % of the total tumor stroma, we defined the staining as “positive.” We further divided positive samples into two groups as follows: “DDAH2-strong” with a positive area stronger than the control evident in 30 % or more of the stroma, and “DDAH2-weak” with a positive area of less than 30 %.

### DDAH2 in situ hybridization

To identify cells that secreted DDAH2, in situ hybridization was performed as reported previously [13]. The details of in situ hybridization are described in the supplementary information.

### Western blot analysis of DDAH2 and eNOS

Protein extraction from surgically resected specimens was performed using T-PER reagent (Pierce, Rockford, IL). Western blotting for DDAH2 was performed using the same protocol as that described above. Western blotting for eNOS was performed as follows. Twenty micrograms of extracted protein was mixed with Laemmli sample buffer, denatured at 95 °C for 5 min, and electrophoresed on 7.5 % Mini Protein TGX gel (Bio-Rad Laboratories, Hercules, CA). Proteins were transferred to polyvinylidene difluoride (PVDF) membranes using an iBlot^™^ gel transfer system (Life Technologies Japan). The membranes were blocked with 0.1 % blocking reagent and then incubated with anti-eNOS antibody at dilution of 1:1000 (ab5589, Abcam) at 4 °C overnight. After washing, immunoreactivity was detected with specific secondary antibodies conjugated to horseradish peroxidase. The protein bands were visualized using Supersignal West Femto maximum sensitivity substrate (Thermo Scientific) and X-ray film (BioMax Light film; Kodak, Rochester, NY).

### Cell culture and reagents

Human umbilical vein endothelial cells (HUVECs) were cultured to evaluate the effect of DDAH2 on angiogenesis in vitro. The details are described in the supplementary information.

### Proliferation and capillary-like tube formation assay

The details of endothelial cell proliferation and capillary-like tube formation assay are described in the supplementary information.

### Capillary-like tube formation assay

To evaluate migration of endothelial cells, we examined capillary-like tube formation by the Geltrex^™^ angiogenesis assay in accordance with manufacturer’s protocol. Briefly, Geltrex^™^ was added to the growth surface and incubated for 30 min at 37 °C to allow the gel to solidify. HUVEC were gently added to each Geltrex^™^ coated 24-well plate at 5 × 10^4^ cells per well in growth medium. Then, DDAH2 recombinant protein or PBS (as control) was added. HUVEC were incubated for 6 h at 37 °C in an atmosphere containing 5 % CO_2_. The cells were visualized directly using a light microscope, and the mean tube length was measured.

### Statistical analysis

Statistical analysis was performed using JMP 9 software. Statistical comparisons between groups were performed using one-way analysis of variance (ANOVA) followed by Student’s *t* test. Disease-free survival (DFS) with DDAH2 expression was compared using the Kaplan-Meier method, and the significance of differences between survival curves was assessed using log-rank test. DFS was determined from the date of surgery until the date of recurrence or last follow-up. Statistical significance was defined as *p* < 0.05.

## Results

### Screening for mouse monoclonal antibodies

**Fig. 1:**
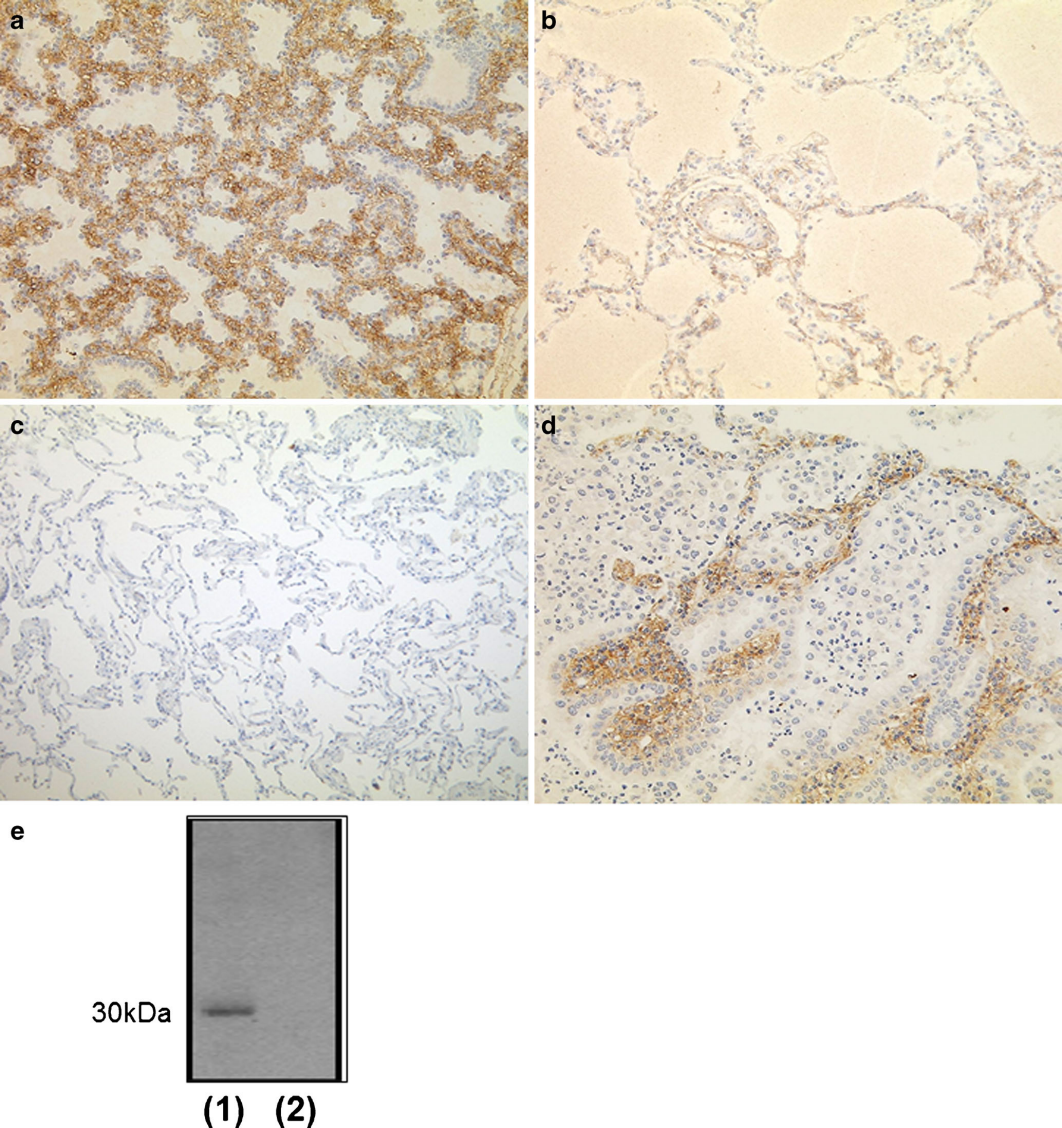
Immunohistochemical screening using culture supernatant of hybridoma clones. Initial screening was performed using swine lung tissues, and we selected clones that positively recognized antigens in fetal miniature swine lung (**a**) and were unreactive with mature swine lung (**b**). In the second screening, normal lung tissue and small lung adenocarcinoma were used and the clones reactive only with lung adenocarcinoma were selected. **c** Absence of staining in normal human lung. **d** Staining of tumor stroma of lung adenocarcinoma. **e** Western blotting using the selected hybridoma supernatant as the primary antibody. Protein bands were detected at approximately 30 kDa with protein extracted from frozen lung adenocarcinoma specimens (*1*), but not from the lung cancer cell line, Calu-3 (*2*)

Ninety-seven hybridoma clones were obtained, and the hybridoma supernatants were used for immunohistochemical screening in two steps. As the first screening, we selected clones that were reactive with fetal but not with mature swine lung (Fig. 1a, b) and obtained 39 clones. We then selected antibodies reactive with lung adenocarcinoma but nonreactive with normal lung tissue, and obtained 11 clones. Of these clones, six showed cytoplasmic staining of cancer cells, but not with normal lung tissue. Four clones showed cytoplasmic staining of cancer cells but also nuclear staining of alveolar type II cells in normal lung tissue. We then immunohistochemically tested these clones on more than 100 adenocarcinomas and found that specificity for lung adenocarcinoma was low. One clone showed specific reactivity with cancer stroma but not with cancer cells (Fig. 1c, d). Because this pattern of reactivity was very characteristic, we selected this clone for further analysis.

### Identification of dimethylarginine dimethylaminohydrolase 2

Using Western blotting, the clone was not reactive with a lung adenocarcinoma cell line (Calu-3) but the sample of lung adenocarcinoma showed a positive band at approximately 30 kDa (Fig. 1e). By immunoprecipitation, we then isolated the recognized antigen in frozen human lung adenocarcinoma tissue, and identified this using LC-MS/MS as DDAH2.

### DDAH2 expression in lung adenocarcinoma

**Fig. 2:**
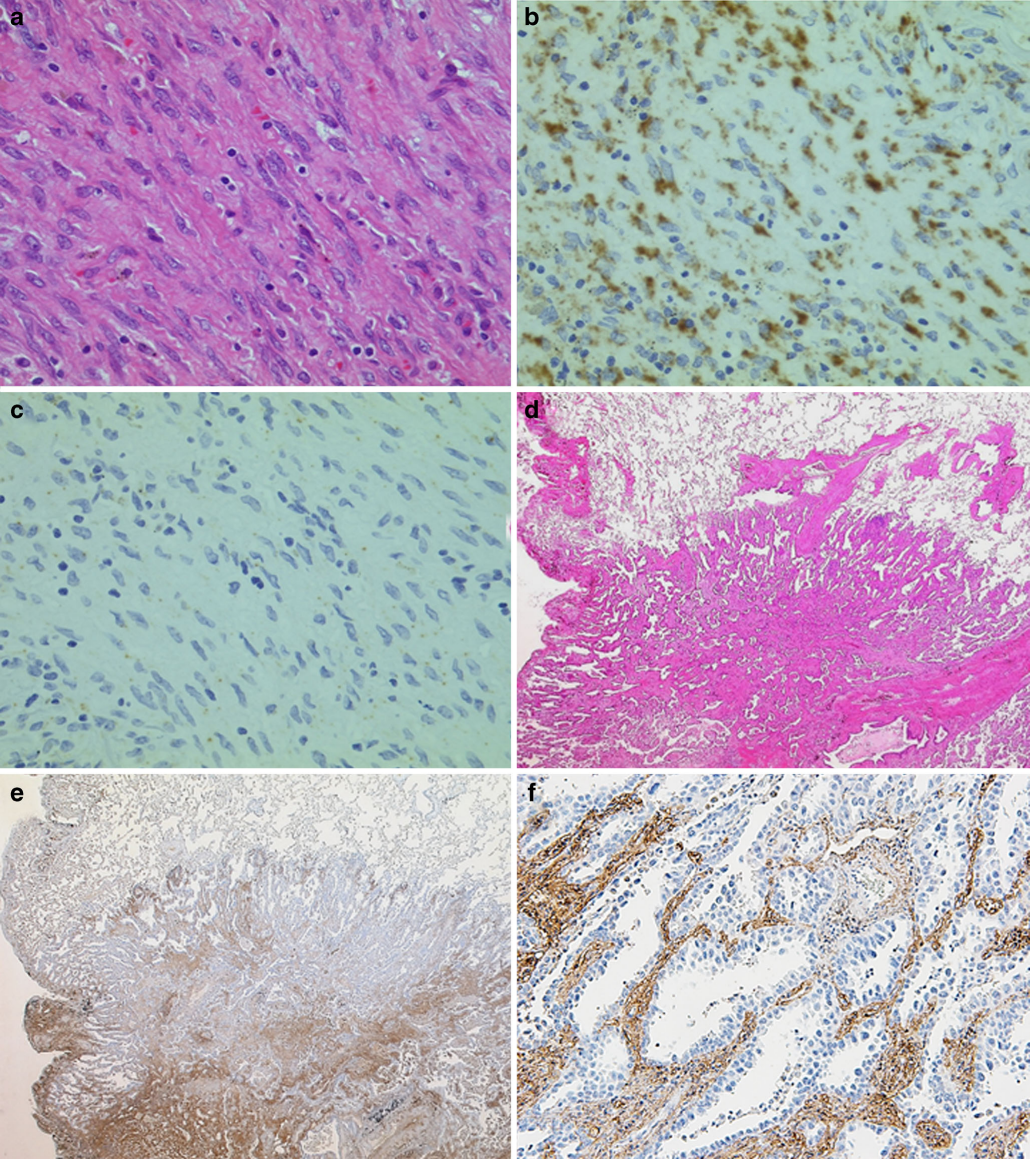
In situ hybridization (ISH) of DDAH2 mRNA using lepidic-predominant invasive adenocarcinoma. **a** Fibroblasts in tumor stroma (HE stain). **b** Fibroblasts are reactive for DDAH2 mRNA with antisense cRNA probe. **c** No reactivity was found when a sense-cRNA probe of DDAH2 mRNA was used. Immunohistochemistry for DDAH2 in lung adenocarcinoma. **d** Representative case of adenocarcinoma showing a lepidic growth pattern. **e** DDAH2-IHC (×10) and (**f**) DDAH2-IHC (×40); the tumor stroma diffusely stains whereas tumor cells are negative

DDAH2 diffusely stained cancer stroma, but the expressing cells could not be identified. Using in situ hybridization, we identified the cells expressing DDAH2 as fibroblasts in the malignant stroma (Fig. 2a–c). Using immunohistochemistry, we then examined expression of DDAH2 on 133 cases of surgically resected lung adenocarcinoma. The stroma of lung adenocarcinoma showed diffuse staining for DDAH2, whereas tumor cells themselves were not stained (Fig. 2d–f). On the other hand, in normal lung tissue, only vascular endothelium showed staining for DDAH2. Table 1 summarizes the proportion of DDAH2-positive cases for each histological subtype of lung cancer. Interestingly, almost all cases of MIA and invasive adenocarcinoma were positive for DDAH2 (MIA 100 %; invasive adenocarcinoma 99 %), while only half of pre-invasive lesions were positive (AAH and AIS 46 %). The staining intensity was not clearly associated with histological evidence of invasion in the pleura or in vessels, or proliferating fibroblasts.

**Table 1:** Proportion of positive cases for DDAH2 immunohistochemistry

Histological subtypes	Positive cases
Preinvasive lesion	21/47 (46 %)
Atypical adenomatous hyperplasia (AAH)	2/14
Adenocarcinoma in situ (AIS)	19/33
Minimally invasive adenocarcinoma (MIA)	11/11 (100 %)
Invasive adenocarcinoma	74/75 (99 %)
Lepidic predominant	40/41
Acinar predominant	7/7
Papillary predominant	8/8
Micropapillary predominant	1/1
Solid predominant	18/18

### DDAH2 expression and patient outcome

**Fig. 3:**
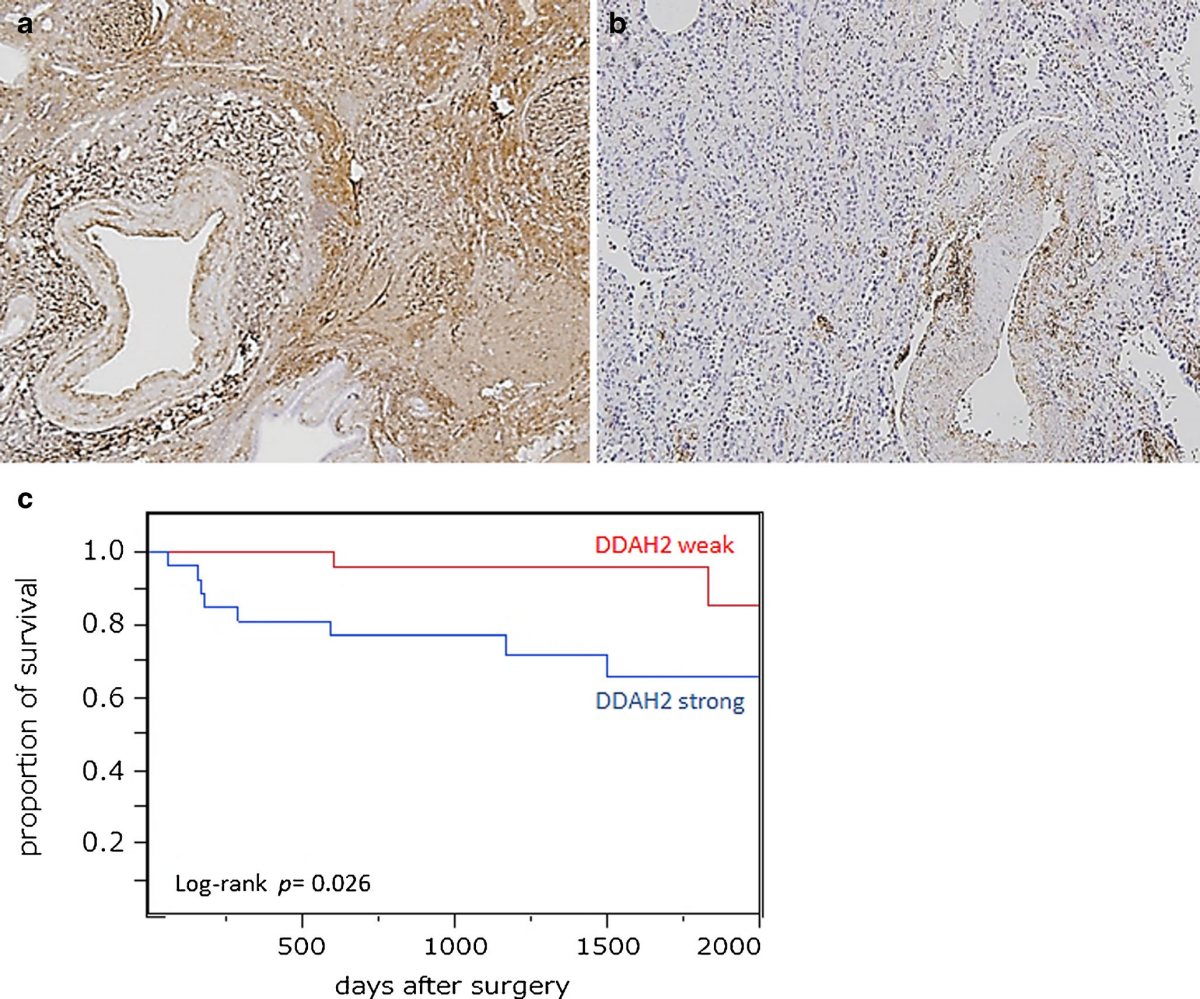
Based on intensity of immunohistochemical staining, 61 cases of stage I adenocarcinoma were divided into (**a**) DDAH2-strong and (**b**) DDAH2-weak. **c** Kaplan-Meier analysis of disease-free survival using log-rank test

We assessed association of DDAH2 expression with patient outcome. We selected 61 pathological stage I cases of which details of the postoperative course were available. The samples were divided into a DDAH2-strong group (Fig. 3a, *n* = 26) and a DDAH2-weak group (Fig. 3b, *n* = 35). The Kaplan-Meier curves showed a significant difference in DFS between the DDAH2-strong and DDAH2-weak groups (*p* = 0.026, Fig. 3c). High expression of DDAH2 was significantly associated with poor outcome.

### DDAH2 and tumor angiogenesis

**Fig. 4:**
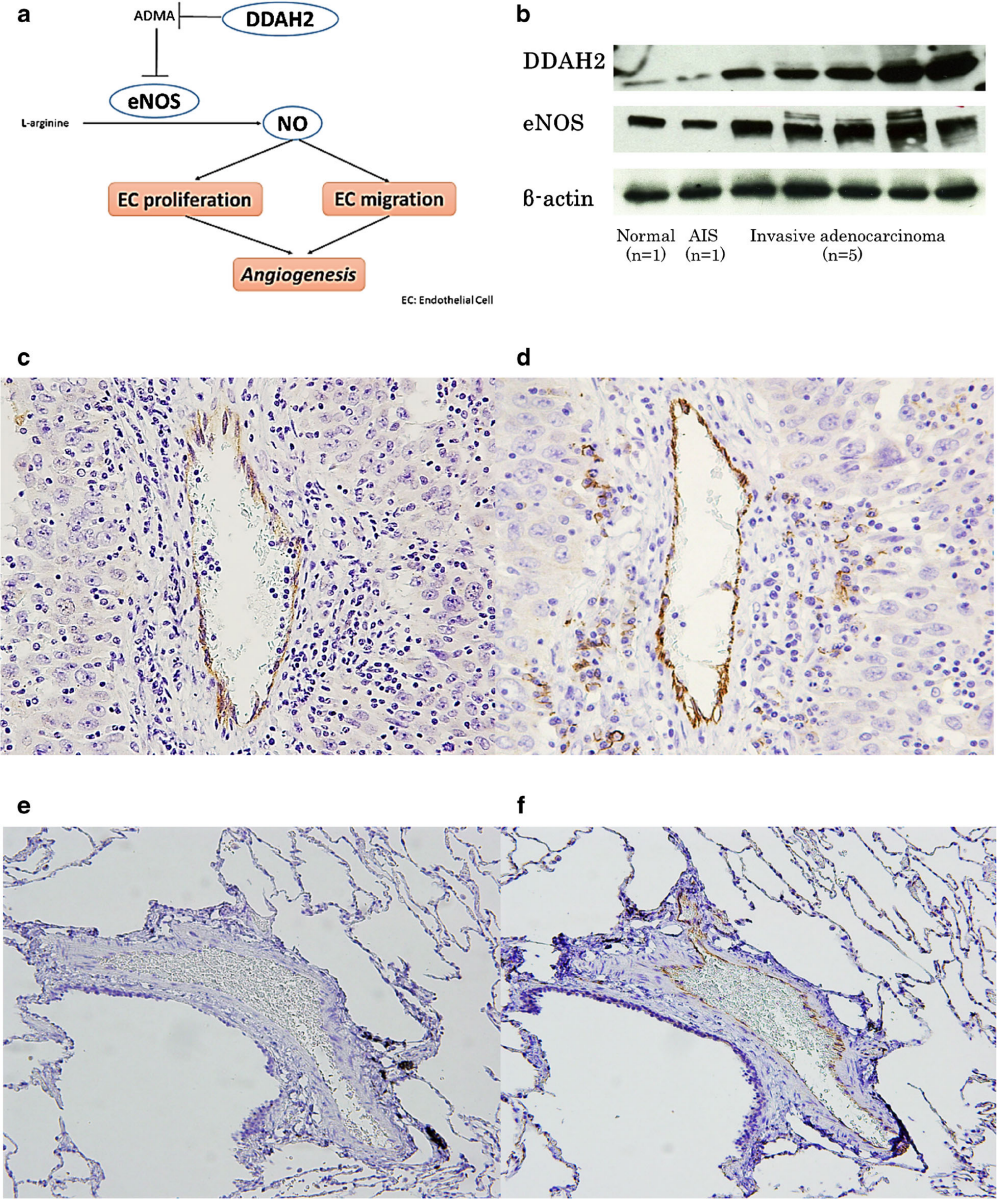
DDAH2 promotes tumor angiogenesis through NO production in lung adenocarcinoma. **a** Tumor fibroblast-derived DDAH2 increases expression of eNOS in vascular endothelial cells, and enhances NO production, followed by upregulation of the kinase cascade. This pathway stimulates endothelial cell proliferation and migration and results in angiogenesis. **b** Samples of normal lung (*n* = 1), AIS (*n* = 1), and invasive adenocarcinoma (*n* = 5) were prepared and used for Western blot analysis with antibodies against DDAH2 and eNOS. Immunohistochemistry with anti-eNOS antibody was performed using the same samples as those used for Western blotting. Vascular endothelial cells were subjected to immunohistochemistry with anti-CD31 antibody. **c** eNOS was expressed strongly in vascular endothelium of the invasive adenocarcinoma. **d** Immunohistochemistry with anti-CD31 antibody in the same section as **c. e** The vascular endothelial cells of normal lung were negative for eNOS. **f** Immunohistochemistry with anti-CD31 antibody in the same section as **e**

We next examined whether DDAH2 might be a prognostic factor for lung adenocarcinoma. We hypothesized that DDAH2 contributes to lung adenocarcinoma invasion through tumor angiogenesis via NO production (Fig. 4a) and expected that eNOS expression might increase upon stimulation by DDAH2. Expression of both DDAH2 and eNOS by Western blotting on seven surgically resected specimens, showed both to be expressed at a significantly higher level in invasive adenocarcinoma than in AIS and normal lung (Fig. 4b). By IHC, we confirmed high expression of eNOS in invasive adenocarcinoma compared to AIS and normal lung, using ten invasive adenocarcinomas, five AIS, and five normal lung tissues. Interestingly, eNOS was expressed strongly in vascular endothelium of malignant stroma (Fig. 4c, d), whereas vascular endothelium of normal lung was negative or showed only faint staining (Fig. 4e, f). These data indicate that DDAH2 stimulated production of eNOS is significantly higher in invasive adenocarcinoma than in AIS and normal tissue.

**Fig. 5:**
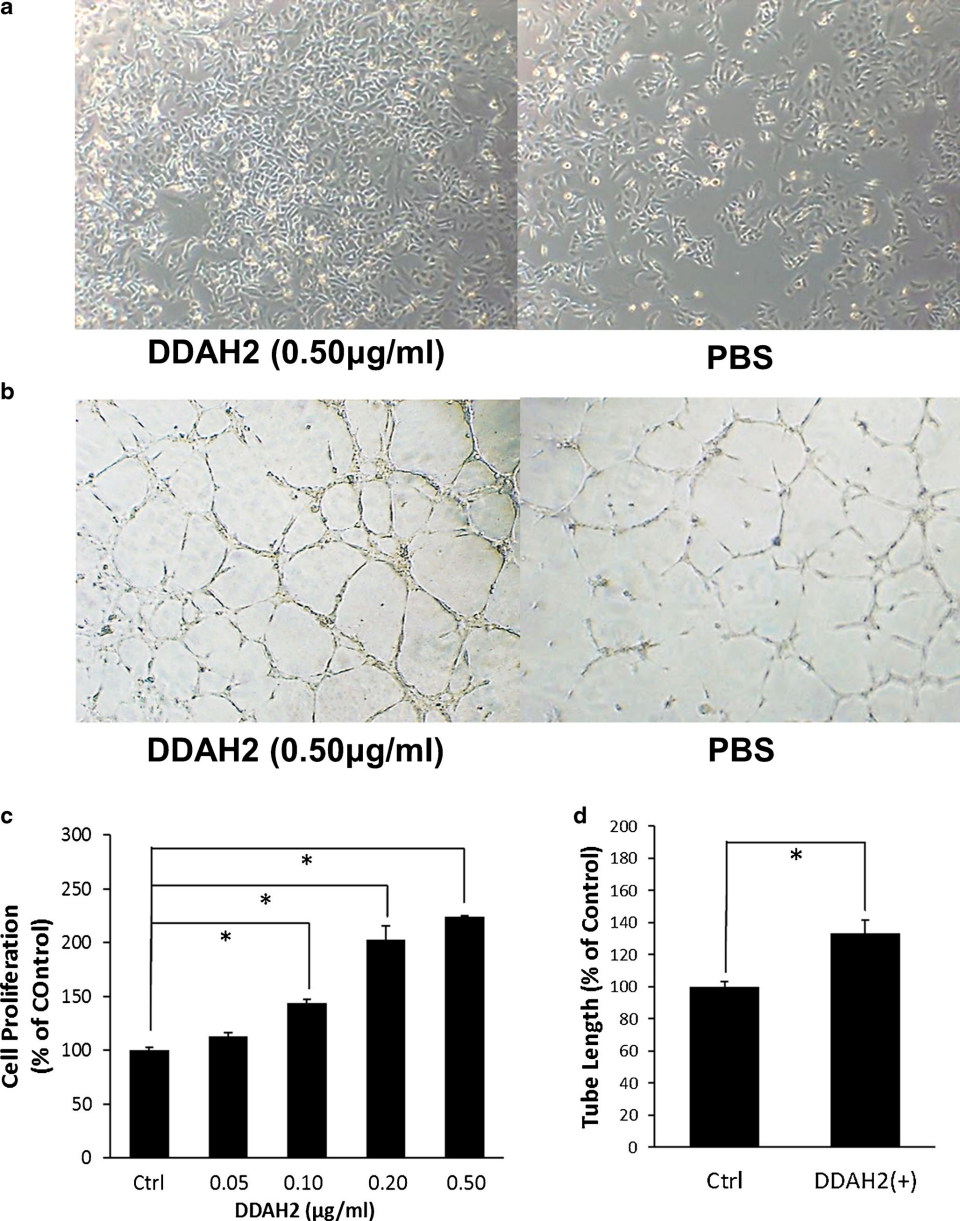
DDAH2 induces proliferation of, and capillary-like tube formation by HUVEC. **a** Recombinant human DDAH2 protein was added to HUVEC at various doses (0.05–0.5 μg/ml) for 48 h. PBS was used as control. **b** Representative photographs illustrate the effects of recombinant human DDAH2 protein on capillary-like tube formation by HUVEC. **c** Cell proliferation was determined by direct counting. **d** The mean tube length in each well was measured. These experiments were performed in duplicate. *p < 0.05

To determine whether DDAH2 regulates angiogenesis through NO production, we next examined the angiogenic effect of DDAH2 in vitro. We first confirmed by Western blotting that DDAH2 recombinant protein significantly enhances eNOS expression in HUVEC (data not shown). We then found that recombinant DDAH2 protein significantly increases the number of HUVEC in a dose-dependent manner (Fig. 5a, c). We also examined the effect of DDAH2 on HUVEC tube formation in an in vitro angiogenesis model. As shown in Fig. 5b, d, DDAH2 recombinant protein significantly enhances capillary-like tube formation in comparison with the control group. Taken together, our findings suggest that DDAH2 promotes tumor angiogenesis in lung adenocarcinoma by increasing the production of NO.

## Discussion

DDAH2 is one of the embryonal proteins expressed in several fetal tissues such as fetal lung and fetal kidney [14]. DDAH2 is expressed mainly in the heart, kidney, and placenta, where it serves as an antiatherosclerotic factor. Asymmetric dimethylarginine (ADMA) is an endogenous inhibitor of eNOS, and DDAH2 metabolizes ADMA to dimethylamine and L-citrulline. DDAH2 activity increases the expression of eNOS, resulting in promotion of vasodilation and vasoprotective activity. In the cardiovascular and renovascular systems, previous studies report that DDAH/ADMA pathway act as a key regulator of angiogenesis through control of endothelial NO production [15, 16].

We developed a monoclonal antibody specifically reacting with stroma of lung adenocarcinoma, and identified the recognized antigen as DDAH2. DDAH2 is one of the embryonal proteins highly expressed in several fetal tissues, including lung and kidney. In hypoplastic human fetal lung, vascular endothelium showed significantly decreased expression of both CD31 and eNOS relative to normal control tissue [17]. It has been suggested that the DDAH2-eNOS pathway plays an important role in the process of angiogenesis in fetal lung. On the other hand, DDAH2 is also regarded as an antiatherosclerotic factor in adults because DDAH2 increases the production of NO, which promotes vasodilation and angiogenesis. Although impairment or decreased expression of DDAH2 has been reported in several diseases such as chronic kidney disease, pulmonary hypertension, and cardiovascular disease [18–22], the association of DDAH2 with malignancy has received less attention.

We initially examined expression of DDAH2 in lung adenocarcinomas using immunohistochemistry and found a significant difference in the proportion of DDAH2-positive cases between MIA, invasive adenocarcinoma, and preinvasive lesions. DDAH2 was expressed in most invasive adenocarcinomas, but only about half of the AIS cases showed DDAH2 staining. Because most lung adenocarcinomas develop in a stepwise manner from AAH to AIS, and then from MIA to invasive adenocarcinoma, it has been suggested that DDAH2 might play an important role in the progression of lung adenocarcinoma, especially in the early phases of tumor invasion. Moreover, we found a statistically significant correlation between the intensity of immunohistochemical staining for DDAH2 and DFS. This suggests that DDAH2 might be a novel prognostic indicator in lung adenocarcinoma and useful for the detection of early invasive adenocarcinoma such as MIA.

We hypothesized that DDAH2 contributes to lung adenocarcinoma invasion through promotion of tumor angiogenesis. As shown in Fig. 4a, expression of tumor fibroblast-derived DDAH2 in vascular endothelium led to an increase of NO production, followed by increased vascular endothelial cell proliferation and migration. Angiogenesis is an important process for tumor growth, and several therapeutic strategies targeting tumor angiogenesis have been developed [23–25]. Only a few previous studies have investigated the function of DDAH in malignant tumors. Kostourou et al. reported that overexpression of DDAH1 is associated with increased neovascularization of gliomas in vivo, and Vanella et al. suggested that DDAH2 might contribute to tumor angiogenesis in prostate cancer through an increase of NO production [26, 27]. Both studies suggest an angiogenic effect of DDAH in malignant tumors. Against this background, we initially examined expression of DDAH2 and eNOS in surgical specimens. eNOS was more highly expressed in invasive adenocarcinoma than in normal tissue or AIS, reflecting the pattern of DDAH2 expression. We then showed that DDAH2 significantly promotes proliferation and capillary-like tube formation of endothelial cells in vitro. These results suggest that tumor angiogenesis in invasive adenocarcinoma is at least partially activated by DDAH2. It is of considerable interest that DDAH2 accelerates the progression of lung adenocarcinoma whereas it works as a preventive factor against cardiovascular and chronic kidney diseases.

One of the noteworthy findings of this study was that DDAH2 is expressed in cancer-associated fibroblasts (CAF) of lung adenocarcinoma. CAF are activated fibroblasts observed in the stroma of various solid tumors. CAF are a major constituent of reactive tumor stroma and play a crucial role in tumor progression and metastasis [28, 29]. Recently, emerging evidence has suggested that CAF also promote tumor angiogenesis in breast and colon cancer by secreting angiogenic factors [30, 31]. In lung adenocarcinoma, previous reports have indicated that CAF play an important role in cancer progression and that their presence is correlated with poor prognosis [32, 33]. However, a role for CAF in angiogenesis in lung adenocarcinoma has not been well characterized. We observed diffuse staining of DDAH2 in cancer stroma and detected expression of DDAH2 mRNA in CAF of lung adenocarcinoma by in situ hybridization. We also found that DDAH2 derived from CAF promotes tumor angiogenesis in lung adenocarcinoma. These results suggest that CAF contribute to tumor angiogenesis in lung adenocarcinoma by secreting DDAH2.

In lung cancer, the angiogenic vascular endothelial growth factor (VEGF) is expressed mainly in tumor cells, and bevacizumab, a humanized anti-VEGF monoclonal antibody, has been developed and is applied as systemic therapy in patients with nonsmall cell lung cancer, with the exception of squamous cell carcinoma [34–36]. However, for control of tumor angiogenesis in lung cancer inhibition of VEGF only is insufficient. Possible functional relationships between VEGF and DDAH2 in lung adenocarcinoma need to be studied. During tumor angiogenesis, VEGF stimulates NO production via activation of the PI3K/Akt cascade, which is distinct from the DDAH/ADMA pathway [37, 38]. Several reports have described a role of DDAH2 in regulating VEGF expression. Hasegawa et al. reported that DDAH2 stimulates expression of VEGF through the transcription factor Sp1 in vascular endothelial cells, and Xiao et al. reported that expression of VEGF mRNA in endothelial cells is significantly suppressed by DDAH2 siRNA [39, 40]. These data suggest that DDAH2 exerts dual effects on NO-mediated angiogenesis. DDAH2 may act not only on vascular endothelial cells by releasing them from ADMA-induced eNOS inhibition but also on tumor cells by stimulating their expression of VEGF. Inhibition of DDAH2 activity in lung adenocarcinoma may therefore become a promising therapeutic strategy complementing conventional antiangiogenic therapy.

We show that DDAH2 plays an important role in tumor angiogenesis in lung adenocarcinoma. However, the mechanism by which DDAH2 expression is regulated in malignant stroma remains unclear. Malignant stroma is an aberrant microenvironment, characterized by persistent hypoxic conditions. Hypoxia is one of the major features of solid tumors, favoring tumor progression and increasing the degree of tumor resistance to treatment [41, 42]. Emerging evidence suggests that hypoxia-inducible signaling pathways are significantly associated with tumor angiogenesis [43, 44]. Among the major transcription regulators involved in the response to hypoxic conditions, hypoxia-inducible factor (HIF) plays an important role in tumor angiogenesis, invasion, and metastasis. HIF promotes tumor angiogenesis through activation of downstream genes, such as VEGF and angiopoetin-2 (Ang-2) [45, 46]. Although the association between hypoxia and DDAH2 is still unclear, DDAH2 might be regulated by HIF, as well as other angiogenic factors. Pullamsetti et al. reported that in murine pulmonary fibrosis transforming growth factor-β1 (TGF-β1) expressed by lung epithelial cells increases the expression of DDAH2 in a time-dependent manner [47]. TGF-β1 is considered to be one of the key regulators of tumor progression, notably in the process of epithelial-mesenchymal transition (EMT) [48]. Several reports have suggested that TGF-β1 also accelerates tumor angiogenesis [49, 50]. These molecules might be potential regulators of DDAH2 in malignant stroma, and further studies aimed at clarifying the mechanism of DDAH2 regulation are needed.

In conclusion, DDAH2 is expressed in CAF in early stage lung adenocarcinoma and might play an important role in tumor invasion by promoting tumor angiogenesis through an increase of NO production. It also might be a novel prognostic factor in lung adenocarcinoma. To elucidate the mechanisms involved in vivo studies will be necessary. The therapeutic potential of DDAH2 as target for inhibition of tumor angiogenesis in lung adenocarcinoma, complementary to conventional antiangiogenic therapy, merits to be explored.

## Electronic supplementary material

ESM 1 (DOCX 17 kb)
