# Supplementary material for: Dimethylarginine dimethylaminohydrolase 2 promotes tumor angiogenesis in lung adenocarcinoma
Source: Virchows Arch. 2015 Oct 29;468:179–90. doi: 10.1007/s00428-015-1863-z (PMC4778151; doi:10.1007/s00428-015-1863-z)
Supplement: Supplementary file 1 — (DOCX 17 kb) [file 428_2015_1863_MOESM1_ESM.docx]

**Supplementary Materials**

**Materials and methods**

1. DDAH2 *in situ* hybridization

To prepare the cRNA probes for *in situ* hybridization of DDAH2, T7 RNA polymerase promoter-attached primers for DDAH2 were designed using the primer designing web tool of Primer 3 (http://primer3.ut.ee/) on the basis of the DDAH2 sequence in the GenBank database (Accession no. NM013974.1). DDAH2 mRNA was amplified by reverse transcriptase–polymerase chain reaction (RT–PCR) with T7 RNA polymerase promoter-attached primers (forward, 5’-CTTAATACGACTCACTATAGGGCTGCTAGAACTGCCACCTGAG-3’; and reverse, 5’-CTTAATACGACTCACTATAGGGGGCTTTGCGGACTCCATCG-3’), and then the PCR products were transcribed to antisense or sense cRNA probes labeled with digoxigenin (DIG) with a DIG RNA Labeling Kit (Roche Diagnostics GmbH, Penzberg, Germany) in accordance with the manufacturer’s instructions. Through this process, we established antisense and sense cRNA probes of about 186 base pairs (bp) for *in situ* hybridization. Formalin-fixed, paraffin-embedded tissue of lepidic predominant invasive adenocarcinoma of the lung was cut into sections 3 μm thick and mounted on silane-coated slides (Matsunami Glass Ind, Osaka, Japan). Hybridization was performed at 50℃ for 16 h in DAKO mRNA In Situ Hybridization Solution (Dako Cytomation, Carpinteria, CA) containing either 0.6 μg/ml heat denatured DIG-labeled anti-sense probe, or the sense probe as a negative control. cRNA probes was performed with horseradish peroxidase-conjugated rabbit anti-DIG antibody (Dako Cytomation, Kyoto, Japan), and the signals were amplified using a GenPointTM Tyramide Signal Amplification System (Dako North America, Carpinteria, CA). Signal detection was performed by immunohistochemical reaction with diaminobenzidine as the chromogen.

1. Cell culture and reagents

Human umbilical vein endothelial cells (HUVECs) were cultured in Ham’s F-12K Medium (Life Technologies Japan) containing 10% fetal bovine serum (FBS). Cells were grown at 37°C in an atmosphere containing 5% CO2. For HUVEC proliferation and capillary-like tube formation assay, DDAH2 human recombinant protein was purchased from Abnova (Walnut, CA), and Geltrex^TM^ was purchased from Life Technologies. Both were stored at -80 ℃ until use.

1. Endothelial cell proliferation assay

HUVEC were seeded in 24-well plates at 1×10^4^ cells per well in F12k medium. The culture medium was exchanged after 24 hours. The cells were then treated with various concentrations of DDAH2 recombinant protein and incubated for 48 hours at 37°C in an atmosphere containing 5% CO2. Cell proliferation was then quantified by cell counting.

1. Capillary-like tube formation assay

To evaluate the migration of endothelial cells, we examined capillary-like tube formation by the Geltrex^TM^ angiogenesis assay in accordance with manufacturer’s protocol. Briefly, Geltrex^TM^ was added at 100μl per well to the growth surface and incubated for 30 min at 37℃ to allow the gel to solidify. HUVEC were gently added to each Geltrex^TM^ coated 24-well plate at 5×10^4^ cells per well in growth medium. Then DDAH2 recombinant protein or PBS (as control) was added. HUVEC were incubated for 6 hours at 37°C in an atmosphere containing 5% CO2. The cells were visualized directly using a light microscope, and the mean tube length was measured.

**Supplementary Table 1**

Clinicopahological characteristics of all cases (n=133)

| Characteristics | | | Number |
| --- | --- | --- | --- |
| age (Av.±SD) | | | 68.7±10.5 |
| gender(M/F) | | | 71 / 62 |
| P-stage |  | I (IA/IB) | 110 (83/27) |
|  |  | II (IIA/IIB) | 12 (9/3) |
|  |  | III (IIIA/IIIB) | 9 (9/0) |
|  |  | IV | 2 |
| Histological | Subtypes | |  |
|  | Atypical adenomatous hyperplasia (AAH) | | 14 |
|  | Adenocarcinoma in situ (AIS) | | 33 |
|  | Minimally invasive adenocarcinoma(MIA) | | 11 |
|  | Invasive adenocarcinoma | |  |
|  |  | Lepidic predominant | 41 |
|  |  | Acinar predominant | 7 |
|  |  | Papillary predominant | 8 |
|  |  | Micropapillary predominant | 1 |
|  |  | Solid predominant | 18 |
